# Association and Genetic Expression between Genes Involved in HPA Axis and Suicide Behavior: A Systematic Review

**DOI:** 10.3390/genes12101608

**Published:** 2021-10-13

**Authors:** Yazmín Hernández-Díaz, Alma Delia Genis-Mendoza, Thelma Beatriz González-Castro, Carlos Alfonso Tovilla-Zárate, Isela Esther Juárez-Rojop, María Lilia López-Narváez, Humberto Nicolini

**Affiliations:** 1 División Académica Multidisciplinaria de Jalpa de Méndez, Universidad Juárez Autónoma de Tabasco, Jalpa de Méndez 86205, Tabasco, Mexico; yazmin.hdez.diaz@gmail.com (Y.H.-D.); thelma.glez.castro@gmail.com (T.B.G.-C.); 2 Laboratorio de Genómica de Enfermedades Psiquiátricas y Neurodegenerativas, Instituto Nacional de Medicina Genómica, Ciudad de México 14610, Mexico; adgenis@inmegen.gob.mx; 3 División Académica Multidisciplinaria de Comalcalco, Universidad Juárez Autónoma de Tabasco, Comalcalco 86650, Tabasco, Mexico; 4 División Académica de Ciencias de la Salud, Universidad Juárez Autónoma de Tabasco, Villahermosa 86150, Tabasco, Mexico; iselajuarezrojop@hotmail.com; 5 Hospital Chiapas “Dr. Jesús Gilberto Gómez Maza”, Tuxtla Gutiérrez 29932, Chiapas, Mexico; dralilialonar@yahoo.com.mx

**Keywords:** stress, genetic, suicide, association

## Abstract

Background: Suicide behavior (SB) has been highly associated with the response to stress and the hypothalamic–pituitary–adrenal (HPA) axis. The aim of this study was to summarize the results obtained in genetic studies that analyzed the HPA axis—stress pathway and SB through a systematic review. Methods: We performed an online search in PubMed, EBSCO, Web of Science, Scopus, and PsycoInfo databases up to May 2021. We followed the PRISMA guidelines for systematic reviews. We included case-control and expression studies that provided data on mRNA expression and single-nucleotide polymorphisms of genes associated with SB. Results: A total of 21,926 individuals participated across 41 studies (not repeats); 34 studies provided data on single-nucleotide polymorphisms in 21,284 participants and 11 studies reported data on mRNA expression in 1034 participants. Ten genes were identified: *FKBP5*, *CRH*, *CRHBP*, *CRHR1*, *CRHR2*, *NR3C1*, *NR3C2*, *SKA2*, *MC2R,* and *POMC*. Conclusions: Our findings suggest that key stress pathway genes are significantly associated with SB and show potential as biomarkers for SB.

## 1. Introduction

Suicide is the act of intentionally ending one’s own life, in which the nonfatal suicidal thoughts and behaviors (hereafter called “suicide behaviors”) are classified specifically into three categories: suicide ideation (SI), suicide plan (SP), and suicide attempt (SA). Suicide ideation and attempts can have negative consequences and are strongly predictive of deaths by suicide [1,2].

Globally, there are approximately 11.4 suicides per 100,000 people, and the suicidality rates are high among those with psychiatric disorders such as depression and anxiety. Moreover, higher rates of SI and SA are observed among females, but higher rates of suicide deaths are observed in males [3,4].

The interaction of internal and external stressors with the psychopathological and cognitive traits form a diathesis for suicide risk [5]. The stress-diathesis model depicts suicide behavior (SB) as a consequence of an interaction between acute stressors and a set of suicide-related traits; this interaction moderates the likelihood of SB in response to stressors [6,7].

**Figure 1:**
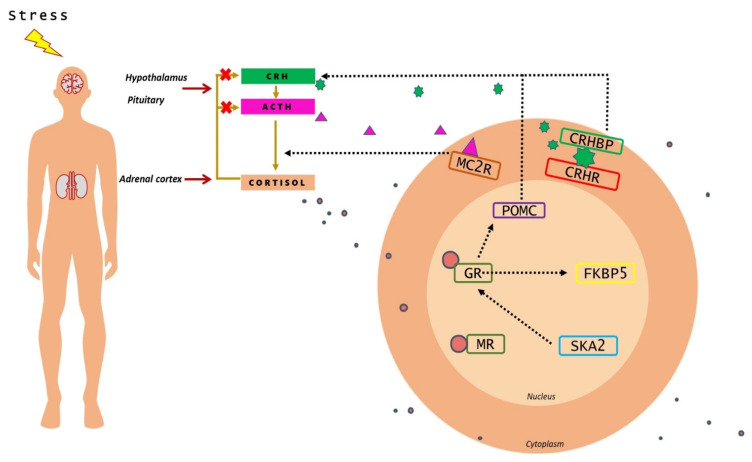
Representative image of HPA axis and the stress pathway genes implicated in suicide behavior. Upon perception of stress, CRH is released from the hypothalamus, which promotes the synthesis and release of ACTH from the pituitary. ACTH, in turn, increases the release of cortisol from the adrenal glands. Through negative feedback, cortisol inhibits the hormone release from the hypothalamus and anterior pituitary glands. Inside the cell, cortisol causes the activation of molecular mechanisms that regulate the impact of the HPA axis.

The response to stress involves a more immediate short-term noradrenergic system response, as well as a more enduring hypothalamic–pituitary–adrenal (HPA) axis response [8]. The HPA system is activated and glucocorticoids (GCs) are released into the systemic blood flow reaching every organ of the body. GCs exert their effects through the mineralocorticoid receptor (MR) and the glucocorticoid receptor (GR), both of which are nuclear receptors [9,10]. When a stressor (either physiological or psychological) is encountered, the hypothalamus releases corticotrophin-releasing hormone (CRH) and vasopressin; when these hormones reach the anterior pituitary, they stimulate the corticotropic cells to release adrenocorticotropic hormone (ACTH). Blood-borne ACTH circulates out of the central nervous system and reaches the adrenal glands above the kidneys, to upregulate the production of GCs, including cortisol (Figure 1).

The ACTH derives from the cleavage of the precursor hormone pro-opiomelanocortin (POMC) by prohormone convertase enzymes. ACTH activates the production and release of cortisol from the zona fasciculata of the adrenal cortex via the melanocortin receptor MC2R [11]. The CRH is regarded as the principal mediator of the stress response in the brain, and its actions are moderated by a high-affinity binding protein (CRHBP) that modulates CRH-mediated activation of CRH receptors in brain and periphery (Figure 1). CRHR1 is the key receptor for CRH-mediated ACTH release in pituitary response to stress. CRHR1 plays a critical role in the acute phase of stress-induced HPA response and CRHR2 is involved in the recovery phase [12,13].

Following stress, cortisol binds to brain tissue with affinity to GRs (encoded by *NR3C1* gene) or MRs (encoded by *NR3C2* gene). MRs are occupied under basal glucocorticoid conditions, whereas GR occupancy is increased as cortisol levels rise following stress. Intracellularly, GR binds to cochaperone FKBP5, an important functional regulator of GR sensitivity; when it is bound to the receptor complex, cortisol binds with lower affinity and nuclear translocation of the receptor is less efficient. *FKBP5* mRNA and protein expression are induced by GR activation; high intracellular levels of FKBP5 lower the binding affinity of GR for glucocorticoids leading to GR resistance [14,15]. The SKA2 protein has been implicated in enabling GR nuclear transactivation [16] (Figure 1), therefore impairing the negative feedback of HPA-axis.

Several studies have demonstrated that stress and a dysregulated HPA axis activity are important additional risk factors of SB [17,18]. Alterations in stress-induced regulation via any genetic factor could have important influences on SB. Some stress pathways genes that have been studied in SB include *NR3C1*, [19,20]; *FKBP5* [21,22]; *CRHR1* [23,24]; and *SKA2* gene [25,26]. Candidate gene studies have shown differentially expressed mRNA patterns and single-nucleotide polymorphisms (SNPs) in Caucasian and African American populations associated with SB [27,28].

Thus, a deep understanding of genetic associations underpinning SB is of paramount importance in developing effective treatment interventions. There is increasing evidence that genes regulating HPA axis have effects on SB.

The dysregulation of HPA axis activity is associated with other systems implicated in suicide, including opioids, serotonin, glutamate systems, lipid status, inflammatory pathways, and neurogenesis; therefore, elucidating the dysregulation of the stress system could help us understand the importance of HPA axis in SB [29,30]. To test this idea, we identified and summarized studies that examined stress pathways genes associated with SB through a systematic review. We included case-control and genetic expression studies that provided data on mRNA expression and single-nucleotide polymorphisms; then, we described the relationship between these genes and SB.

## 2. Methods

### 2.1. Search Strategy

A search for studies that investigated the association between HPA axis—stress pathway genes and SB was conducted up to May 2021 following the Preferred Reporting Items for Systematic Reviews and Meta-Analyses (PRISMA) guidelines (Supplementary Table S1). The systematic search was performed using five online databases: PubMed, EBSCO, Science Direct, PsychInfo, and Scopus in order to find and include the most pertinent literature.

Keywords included in the literature search were: (1) for SB: suicide, suicide plan, suicide ideation, suicide attempt; (2) for stress: HPA, cortisol, stress pathway; and (3) for genetic influences: gene, genotype, SNP, polymorphism, gene expression, and candidate gene. The scope of the online search was further expanded by assessing bibliographic references of the eligible full text articles in order to detect other relevant studies.

### 2.2. Inclusion Criteria and Data Extraction

The studies were initially retrieved as title and abstract and screened for eligibility. To be selected, the articles had to fulfill the inclusion criteria: (1) original article, (2) peer-reviewed research, (3) articles published between 2001 and 2021, (4) case-control and expression studies that provided data on mRNA expression and single-nucleotide polymorphisms of stress pathway genes associated with SB, and (5) to be written in English.

After removing duplicates and scanning titles and abstracts, articles that met the inclusion criteria were reviewed. The following data were extracted from each eligible article: authors, year of publication, studied population (number of participants, ethnicity, diagnostic), tissue source, gene name, polymorphism, gene expression, and main findings of the study. Two authors (YHD and TBGC) conducted all screening analysis and data extraction.

### 2.3. Quality Assessment of Primary Studies

The methodological quality of the included studies was evaluated using the Newcastle–Ottawa scale (NOS). The NOS scale gives scores that range from zero to nine, giving a point to each accomplished item and categorizing the studies as high quality (score 7–9), moderate quality (score 4–6), or poor quality (score 0–3). The tool assesses the studies based on three dimensions: selection, compatibility, exposure, or outcome. Authors rated the article independently and discussed the ratings.

### 2.4. Data Synthesis

Significant information from the studies included was carefully organized; the phenotypic outcomes that were considered as SB in this systematic review were: suicide ideation (SI), suicide attempt (SA), suicide plan (SP), and completed suicide (CS). The most promising genes were extracted from the results and the main findings from the texts; tables summarized the study characteristics.

## 3. Results

### 3.1. Study Selection

**Figure 2:**
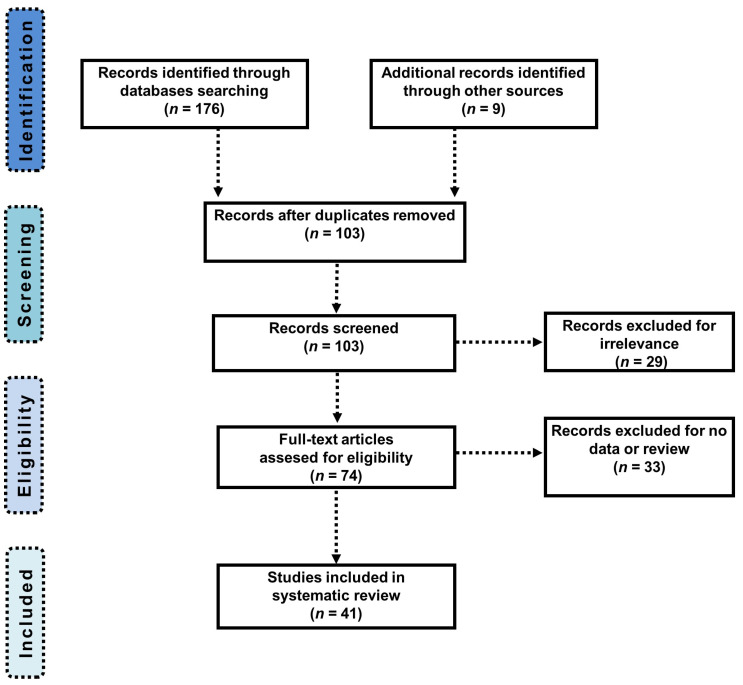
PRISMA flow chart presenting the articles identification and selection processes.

Figure 2 highlights the identification and selection process following the PRISMA statement. The search in PubMed, EBSCO, Science Direct, PsychInfo, and Scopus databases resulted in a total of 176 identified articles, and 103 articles remained after removal of duplicate records. Then, 62 unrelated articles were excluded; finally, 41 articles were accepted for the systematic review based on our inclusion and exclusion criteria. The analysis outcomes of the selected publications are shown in Table 1 and Table 2.

### 3.2. Studies Caracteristics

A total of 21,926 individuals (repeated individuals were excluded) participated across the 41 studies. Thirty-three studies included a control group and 8 studies only evaluated cases. In the majority of studies, controls were described as healthy individuals. The sample sizes ranged from 7 to 3623. All the studies were conducted between 2001 and 2021.

The main psychiatric disorders present in individuals with SB were major depression disorder, bipolar disorder, and substance use dependence. Tissue sources utilized for genotyping or genetic expression analyses were blood, brain, saliva, and buccal cells. The methods used for measuring trauma exposure also differed across studies, including the childhood trauma questionnaire (CTQ), early life events scale (ELES), life events checklist (LEC), and the adverse childhood experiences questionnaire (ACE). A comprehensive description of the studies characteristics is presented in Table 1 and Table 2. Finally, the quality assessment using NOS scale revealed a mean score of 7.17 (ranging from 6 to 9) for SB studies (Table 3).

### 3.3. Phenotypes and Genes

Ten genes were identified: *FKBP5, CRH, CRHBP, CRHR1, CRHR2, NR3C1, NR3C2, SKA2, MC2R,* and *POMC.* Of the included studies that analyzed SNPs in SB, SA was the phenotype most frequently evaluated, followed by SI and CS. In total, 264 DNA SNPs comprised in 10 different genes were analyzed across the studies included in this review (Table 1).

We observed an upregulation of *CRH* and *SKA2* genes; however, findings on mRNA expression were not consistent across studies, as some studies indicated a downregulation of *SKA2* or did not find important changes. The *NR3C1, FKBP5, CRH1,* and *SKA2* genes were the most frequently studied in expression studies (Table 2). Finally, CS and SI were the phenotypes most evaluated in the studies that analyzed mRNA/gene expression levels.

### 3.4. Synthesis of Results

#### 3.4.1. *FKBP5* Gene

Eighteen studies [19,21,22,28,31,32,33,34,35,36,37,38,39,40,41,42,43,44] analyzed the association between *FKBP5* SNPs and SB phenotypes comprising 4239 cases and 9646 controls. The first study conducted in 2007 by Papiol et al. [31] highlighted a significant association between the rs1360780 SNP and SA. Significant associations were also identified between rs3800373, rs3777747, rs2766533, rs4713902, rs9470080, rs1043805, and rs9296158 SNPs and SB in other studies. Additionally, rs3800373 was significantly associated with stress exposure.

On the other hand, mRNA expression levels and SB were analyzed in five studies [14,41,56,57,58] including 204 cases and 145 controls. The *FKBP5* gene was downregulated in samples of brain and blood, as well as mRNA which was consistently correlated with heroin, painkillers, and ecstasy use [58].

#### 3.4.2. *CRH* Gene

Four studies [27,28,38,45] examined the *CRH* SNPs and suicide attempt, including 1395 cases and 1496 (Table 1). All the studies reported that SNPs in this gene were not significantly associated with SA.

Only two studies [55,60] analyzed the expression levels of *CRH* gene in CS including 29 cases and 19 controls. Both studies demonstrated that *CRH* gene was upregulated in brain tissue of CS compared with brain tissue from controls.

#### 3.4.3. *CRHBP* Gene

Five studies [19,27,28,31,38] investigated the *CRHBP* SNPs, each with significant findings (1016 cases and 1592 controls). In 2010, De Luca et al. [27] observed that the heterozygous genotype of rs1875999 was significantly associated with SA and risk of SA. Additionally, Roy et al. [28] found that rs6453267, rs7728378, and rs10474485 showed a nominally significant interaction with the continuous CTQ score to predict SA. No changes in the *CRHBP* gene expression between cases and controls were observed [55,56,60].

#### 3.4.4. *CRHR1* Gene

Sixteen studies [19,23,24,27,28,31,38,42,44,45,46,47,48,49,50,51] evaluated the association between the *CRHR1* SNPs and SB phenotypes in 3718 cases and 4539 controls. Significant associations were identified between s7209436, rs110402, rs16940665, rs4792887, rs12936511, rs1396862, rs878886, and rs242948 SNPs and SB.

In a study conducted by Pawlak et al. [49], the rs16940665 polymorphism was associated with males who had attempted suicide and had major depression disorder. Ludwig et al. [51] indicated that there was a significant gene-environment-interactions for rs7209436 and rs110402 SNPs, reflecting the impact of childhood trauma and *CRHR1* polymorphisms on previous SA.

Four studies [55,56,59,60] analyzed the *CRHR1* gene expression levels in CS and SI (52 cases and 46 controls). A *CRHR1* downregulation was observed in only one study associated with CS [60].

#### 3.4.5. *CRHR2* Gene

Seven studies [19,23,27,28,31,38,47] evaluated the association between *CRHR2* SNPs and SB in 1407 cases and 1775 controls. Allele G carriers of rs2270007 showed a worse overall response to citalopram in follow-up time and showed a 2.93 increased risk of nonresponding to citalopram at week 4 of treatment. Additionally, sexual abuse in childhood and childhood emotional neglect interacted with the rs255098 to modulate adult decision making in SA [31,47].

Three studies [55,59,60] reported no changes in the mRNA levels of this gene in CS (38 cases and 26 controls).

#### 3.4.6. *NR3C1* Gene

Eight studies [19,20,27,36,38,41,44,48] (783 cases and 2570 controls) analyzed the association between *NR3C1* SNPs and SB phenotypes. The first one was conducted in 2010 by De Luca et al. [27] and highlighted a significant association between rs6196 SNP and SA. Other associations were identified between rs9324924, rs2963155, and rs41423247 SNPs and SB in other studies.

mRNA expression levels and SB were analyzed in eight studies [14,41,53,54,55,56,57,58] including 186 cases and 198 controls. *NR3C1* gene was observed to be downregulated in samples of brain and blood. mRNA was significantly reduced in individuals who CS, many of whom had history of childhood abuse in comparison with non-abused CS [53]. Cortisol levels were associated with mRNA [57] and with the expression levels of rs10052957, rs6190 and rs41423247 SNPs [54].

#### 3.4.7. *NR3C2* Gene

Two studies [19,36] examined the *NR3C2* SNPs and SB in 348 cases and 228 controls. All studies reported that SNPs in this gene were not significantly associated with SB. No data were reported for expression.

#### 3.4.8. *SKA2* Gene

Four studies [25,41,44,52] examined the association between *SKA2* SNPs and SB in 1675 cases and 1320 controls. rs8082544 and rs7502947 showed an association with CS [41] as well as significant interactions for *SKA2* 3′-UTR DNA methylation, while the rs7208505 SNP was associated with SI and SA [25].

Four studies [26,41,57,58] analyzed *SKA2* gene expression levels and CS in 179 cases and 193 controls. Data showed a downregulation of *SKA2* gene; however, findings in mRNA expression were not consistent across studies, as some studies indicated an upregulation of *SKA2* or no changes [57,58]. No associations with trauma exposure and concentrations of cortisol were indicated.

#### 3.4.9. *MC2R* Gene

Three studies [19,27,38] evaluated the association between the *MC2R* SNPs and SB phenotypes in 841 cases and 807 controls. All studies reported that the SNPs in this gene were not significantly associated with SB. No data were reported for expression.

#### 3.4.10. *POMC* Gene

Two studies [19,38] investigated the *POMC* SNPs (in 760 cases and 657 controls) in association with SB. Both studies reported that this gene was not associated with SB and no data were reported for expression.

## 4. Discussion

This systematic review aimed to summarize the findings of genetic variants that have been associated with SB. We reviewed 41 publications that gathered 10 promising genes associated with SB: *FKBP5, CRH, CRHBP, CRHR1, CRHR2, NR3C1, NR3C2, MC2R, SKA2,* and *POMC*.

### 4.1. Main Findings

The study of polymorphisms may contribute, at least in part, to explain the alterations observed in SB; additionally, different polymorphisms could alter the genes expression levels and HPA activity in response to stress [14,56]. Our results are in agreement with studies that utilize others approximation. A recent study using a network meta-analysis observed that *FKBP5* gene in union with other mediators could increase the risk of suicide behavior [61]. Additionally, studies suggest that these mediators could be childhood victimization [21]. They found that *FKBP5, CRHBP,* and childhood victimization could increase the risk for suicide behavior. Additionally, several studies indicated that genetic and epigenetic variations in different regions of *FKBP5* gene may contribute, at least in part, to the *FKBP5* alterations observed in SB. Then, the positive evidence in the literature and our results in the present systematic review suggest a possible role of *FKBP5* gene in suicidal behavior.

Second, we found that other genes such as the CRH family *(CRH, CRHR1,* and *CRHR2* genes) and *CRHBP* gene (an antagonist of the stress hormone CRH) showed conflicting results between SNPs and mRNA expression levels. As an example, in the frontopolar cortex, mRNA for *CRHR1*, but not *CRHR2* receptors were reduced in brains of individuals who died by suicide, possibly secondary to high levels of CRH activity [60]. This could be partially explained by ethnic discrepancies or studies with small sample sizes observed in the studies.

Third, our findings suggest that GR (encoded by *NR3C1*) might underlie a contribution of HPA axis to SB phenotypes. Functional polymorphisms within the *NR3C1* gene may impact its gene expression [54]; moreover, mRNA was positively and moderately correlated with hair cortisol concentrations and also negatively correlated with childhood abuse [57]. However, we observed that *NR3C2* gene (mineralocorticoid receptor) did not play a role in SB [19].

Fourth, no significant associations between *MC2R* and *POMC* genes with SB were reported. Alternatively, polymorphisms in these genes might be in high linkage disequilibrium with the causative variants. Studies have shown that epigenetics, especially DNA methylation, play an important role in the occurrence, development, and progression of psychiatric disorders. In addition, research on epigenetics proves that environmental factors are also closely related to the occurrence of diseases [62,63]. Nonetheless, literature on these genes is extremely poor, and, therefore, further research is required to confirm or reject the hypothesis of their non-association with SB.

Fith, while most of the articles examined focused on one or a few candidate genes, SB is a complex and polygenic disease with each genetic variant likely to be contributing a small percentage to disease. Then, studies the GWAS studies that analyze the specially the genes implicated in the HPA axis are necessary.

Finally, we observed a variation across studies in terms of psychiatric disorders and exposure to traumatic events. Individuals with serious mental illnesses (e.g., schizophrenia, bipolar disorder, major depressive disorder) have significantly higher suicide rates than the general population; additionally, the heterogeneity of the findings could indicate that the presence of a mental illness as well as the expression of genetic and environmental effects (traumatic events) could contribute to different phenotypes. This also highlights the importance of conducting psychiatric diagnostic stratified studies.

### 4.2. HPA Axis and Suicide Behavior

A dysfunction of the hypothalamic-pituitary-adrenal (HPA) axis is considered a possible pathogenic background of suicide. Because some polymorphisms regulate the genic expression levels that lead to GR resistance and impaired negative feedback, we could speculate that some alleles cause a slower return to baseline of stress-induced cortisol levels, increasing the risk for psychiatric disorders such as SB. As gene expression is responsive to cortisol, genetic modifications that alter this interaction could modulate the effects of environmental stressors on HPA axis [28,44].

Altered mechanisms may exert deleterious effects on the development of brain structures implicated in suicide behavior. In both of these contexts, genes may contribute to alter neurobiological functions, and a maladaptive prolonged stress response may render individuals more vulnerable to suicide [18]. The specific pattern of this intracellular crosstalk may vary across tissues and may contribute to the pleiotropic consequences of HPA axis dysregulation in suicide [64,65]. Therefore, elucidating the molecular underpinnings of this variability is of great relevance for developing individualized prevention strategies and treatments for individuals with SB. Finally, drugs targeting the function of HPA axis genes may potentially serve to prevent negative long-term effects of stress.

### 4.3. Strengths and Limitations

This is the first systematic review to explore the association between stress pathways (particularly the HPA axis) genes and SB. While some methodological weaknesses were observed, most studies were well designed and conducted according to the NOS scale. Nonetheless, this systematic review has some limitations. Findings within this review were at times conflicting. Incongruities may be partly explained due to the differences in methodological aspects such the participant characteristics. For example, the presence of a psychiatric disorder, current use of medication, and differences in the racial/ethnic component may affect the susceptibility to SB. Suicide is a complex disease involved in the regulation of a series of genetic factors besides HPA axis genes. As a multifactorial disease, the risk of developing it is closely related to various elements, and not just a single factor. Second, exposition to adversities during childhood influence the development of SB; however, several studies not taken this characteristic into consideration. Third, there is a lack of endophenotype data that may help to understand the association between genes and SB. Another drawback was that several studies examined a small sample population, and many did not establish statistical significance due to this. Finally, we cited articles written in English only, thus we could have missed important articles in other languages.

### 4.4. Future Directions

Future research studies should focus on the simultaneous analysis of the widest possible range of genes and their interactions. It is important to consider epigenetic variation of gene activity that can occur as a reaction to external factors. Populations should be divided by sex, as SB is different between females and males. Further still, more extensive explorations of the candidate genes highlighted in this review should provide further insight into the pathogenesis of suicide behavior.

## 5. Conclusions

This review identified and systematically compiled key stress pathways (particularly the HPA axis) genes that are significantly associated with SB. In total, 10 genes that predicted suicide risk were identified. The outcomes of this review could help to further illuminate the genetic basis of suicide behavior. Further research into this field is definitely necessary to achieve a better understanding of the pathogenesis of SB phenotypes.

## Figures and Tables

**Table 1:** Detailed analysis of the selected publications regarding the association between single-nucleotide polymorphisms (SNP) in HPA genes and the pathogenesis of suicide behavior.

Author, Year	Chromosome	SNP	Location	Substitution	Diagnostic	Suicide Behavior	Population Data (N)		Measurement on Trauma Exposure	Tissue Source	Main Outcomes
							*Cases*	*Control*			
*FKBP5*											
Papiol, S. 2007 [31]	6	rs1360780	Intron	T > C	MDD	SA	24	96	-	Blood	T allele carriers showed 2.10 increased for non-responding to citalopram treatment at week 4
Willour, V. L. 2009 [32]	6	rs1043805	3’UTR	T > A	BD	SA	544	-	-	-	Four SNPs showed significant associations with SA: rs1043805, rs3800373, rs9296158 and rs1360780.
		rs3800373	3′UTR	C > A							
		rs7757037	Intron	G > A							
		rs3798346	Intron	A > G							
		rs9296158	Intron	G > A							
		rs1360780	Intron	T > A							
		rs4713902	Intron	T > C							
		rs6912833	Intron	A > T							
		rs9380525	Intron	G > A							
Brent, D. 2010 [33]	6	rs3800373	3′UTR	C > A	MDD	SB	18	-	-	Blood and Buccal	Genotypes of rs1360780TT and rs3800373GG were associated with SB, even after controlling for treatment effects and relevant covariates.
		rs1360780	Intron	T > A							
Roy, A. 2010 [34]	6	rs3800373	3′UTR	C > A	SUD	SA	248	1465	CTQ	Blood	1. Three SNPs showed significant associations with SA: rs3777747, rs4713902, and rs9470080. 2. Three SNPs showed a G X E interaction: rs3800373, rs9296158, and 1360780. 3. There were no interactive effects between substance dependence, CTQ scores, and *FKBP5* SNPs in SA.
		rs9470065	Intron	G > A							
		rs9296158	Intron	G > A							
		rs4713899	Intron	G > A							
		rs9470067	Intron	G > A							
		rs3777747	Intron	G > A							
		rs7762668	Intron	G > A							
		rs9462099	Intron	T > C							
		rs9380524	Intron	C > A							
		rs1360780	Intron	T > C							
		rs7771722	Intron	G > A							
		rs4713902	Intron	T > C							
		rs9462100	Intron	T > C							
		rs2092427	Intron	G > A							
		rs7762760	Intron	G > A							
		rs9470080	Intron	T > C							
Perroud, N. 2011 [35]	6	rs1360780	Intron	T > C	DE	SI	131	-	-	-	The T allele was a risk factor of SI and it was associated with the response to antidepressant treatment.
Supriyanto, I. 2011 [36]	6	rs3800373	3′UTR	C > A	SV	CS	219	228	-	Blood	No association.
		rs1360780	Intron	T > C							
		rs2395635	Intron	A > C							
Roy, A. 2012 [28]	6	rs3800373	3′UTR	C > A	SUD	SA	141	689	CTQ	Blood	1. In the group exposed to severe trauma, the prevalence of SA was 0.49 in carriers of the major homozygote. 2. An analysis of the interaction of total CTQ score with combined *FKBP5* rs3800373 and *CRHBP* rs7728378 genotypes was significant.
Leszczynska-Rodziewicz, A. 2014 [37]	6	rs1360780	Intron	T > C	BD	SA	156	724	-	Blood	No association.
		rs755658	Intron	C > T							
		rs4713916	Intron	A > G							
		rs7748266	Intron	T > C							
		rs9296158	Intron	G >A							
		rs9394309	Intron	G >A							
		rs9470080	Intron	T > A							
		rs3800373	3′UTR	C > A							
Breen, M. E. 2015 [38]	6	rs6926133	Intron	A > C	BD	SA	631	657	ELES	-	No association.
		rs12200498	Intron	G > A							
		rs9380526	Intron	C > T							
		rs16879378	Intron	A > C							
		rs4713899	Intron	G > A							
Fudalej, S. 2015 [39]	6	rs3800373	3′UTR	C > A	SV	CS	520	475	-	Blood	A significant association between the high-induction rs3800373 C allele and SV was detected.
		rs1360780	Intron	T > C							
Breen, M. E. 2016 [40]	6	rs141713011	Intron/3′UTR	G > T	BD or SD	SA	476	473	-	-	rs141713011 showed an excess of minor alleles in SA that was statistically significant following correction for multiple testing, but it could not be replicated.
		rs140664762	Intron/3′UTR	G > A							
		rs575259136	Intron	A > AAAG							
		rs13192954	Intron	A > G							
		rs553156199	3′UTR	C > T							
Yin, H. 2016 [41]	6	rs3800373	3′UTR	C > A	MDD	SA and CS	SA: 87 CS: 121	SA: 190 CS: 88	-	SA: - SV: Brain	1. rs9296158, rs3777747, rs4713902, rs7757037, rs737054, and rs9380529 showed evidence of association at uncorrected *p* < 0.05 level with SA 2. There was no evidence of an association between these SNPs and death by suicide in the postmortem sample.
		rs9296158	Intron	A > C							
		rs3777747	Intron	A >G							
		rs4713902	Intron	T > C							
		rs9470080	Intron	T > A							
		rs7757037	Intron	G > A							
		rs737054	Intron	G > A							
		rs9380529	Intron	A > C							
Mirkovic, B. 2017 [42]	6	rs1360780	Intron	T > C	Mixed	SA	98	150	-	Saliva	No association.
		rs3800373	3′UTR	C > A							
Segura, A. G. 2019 [19]	6	rs3777747	Intron	A >G	BD	SB	129	-	CTQ	Blood	1. rs3777747AA and rs2766533GG genotypes were associated with SB. 2. Did not find an interaction between any CTQ scores and SNPs.
		rs1360780	Intron	T >A							
		rs17542466	Intron	A > C							
		rs2766533	Intron	G > A							
Zhang, L. 2019 [43]	6	rs1360780	Intron	T > C	PTSD and DE	SI	266	3623	LEC	Saliva	No association.
		rs9470080	Intron	T > A							
		rs3800373	3′UTR	C > A							
		rs9296158	Intron	A > C							
Nobile, B. 2020 [44]	6	rs737054	Intron	G > A	MDD	SI and SA	SI: 99SA:9	384	-	Buccal	The TT genotype of rs737054 and TT genotype of rs6902321 were significantly associated with SI. These associations were not significant after multiple test corrections.
		rs6902321	Intron	C > T							
		rs3800373	3′UTR	C > A							
		rs7757037	Intron	G > A							
		rs1360780	Intron	T > C							
		rs9470080	Intron	T > A							
Berent, D. 2020 [21]	6	rs1360780	Intron	T > C	AD	SA	176	127	ACE	Buccal	No association.
Hernández-Díaz, Y. 2021 [22]	6	rs4713916	Intron	A > G	SA	SA	146	277	-	Blood	1. rs1360780 T minor allele was found to be a risk factor for SA. 2. rs3800373 C minor allele was found to be a protective factor for SA.
		rs1360780	Intron	T > C							
		rs4713902	Intron	T > C							
		rs3800373	3′UTR	C > A							
		rs9296158	Intron	A > C							
*CRH*											
Wasserman, D. 2008 [45]	8	rs1870393	Intron	A > C	Mixed	SA	542	-	SLEI	Blood	No association.
		rs3176921	5′ region	C > T							
De Luca, V. 2010 [27]	8	rs3176921	5′ region	C > T	SZ	SA	81	150	-	Blood	No association.
Roy, A. 2012 [28]	8	rs6996265	Intron	A > G	SUD	SA	141	689	CTQ	Blood	No association.
		rs3176921	5′ region	C > T							
		rs6472257	5′ region	C > T							
		rs5030875	Intergenic	T > G							
Breen, M. E. 2015 [38]	*8*	rs6990486	Downstream	G > A	BD	SA	631	657	ELES	-	No association.
		rs6472257	Upstream	C > T							
		rs7835214	Downstream	T > C							
		rs10957368	Downstream	T > C							
		rs10105164	Downstream	C > T							
*CRHBP*											
Papiol, S. 2007 [31]	5	rs7728378	Intron	C > T	MDD	SA	24	96	-	Blood	No association.
		rs1875999	3′UTR	A > G							
De Luca, V. 2010 [27]	5	rs1875999	3′UTR	A > G	SZ	SA	81	150	-	Blood	The heterozygous genotype was significantly associated with SA as a risk of attempt.
Roy, A. 2012 [28]	5	rs3792738	5′UTR	C > A	SUD	SA	141	689	CTQ	Blood	rs6453267, rs7728378, and rs10474485 showed a nominally significant interaction with the continuous CTQ score to predict SA. 2. There was an additive effect of *FKBP5* rs3800373 and *CRHBP* rs7728378 in the group exposed to severe trauma.
		rs328967	Intron	A > G							
		rs6453267	Intron	G > A							
		rs7728378	Intron	C > T							
		rs1875999	3′UTR	A > G							
		rs10474485	Intron	C > A							
		rs1715747	Intron	C > T							
		rs1500	Alt isoform	C > G							
Breen, M. E. 2015 [38]	5	rs7721799	Intron	G > A	BD	SA	631	657	ELES	-	No association.
		rs2174444	Downstream	C > T							
		rs10473984	Downstream	G > T							
Segura, A. G. 2019 [19]	5	rs7728378	Intron	C > T	BD	SB	139	-	CTQ	Blood	1. rs7728378-C carriers were associated with SB. This association did not remain significant after correcting for multiple comparisons. 2. Did not find an interaction between any CTQ scores and SNPs.
		rs10474485	Intron	C > A							
*CRHR1*											
Papiol, S. 2007 [31]	17	rs110402	Intron	C > T	MDD	SA	24	96	-	Blood	TT homozygous had nearly 3 times more risk to develop seasonal pattern episodes.
		rs242937	Intron	A > C							
Wasserman, D. 2008 [45]	17	rs1396862	Intron	G > A	Mixed	SA	542	-	SLEI	Blood	Stratification based on the levels of lifetime stress showed reproducible association and linkage of rs4792887 to SA exposed to low levels of stress mainly in males who were depressed.
		rs4792887	Intron	C > T							
Wasserman, D. 2009 [24]	17	rs4792887	Intron	C > T	Mixed	SA	672	-	SLEI	Blood	1. The minor T-allele of rs12936511 was significantly transmitted in males with SB and with increased BDI scores. 2. Association and linkage with increased BDI scores among suicidal males with an additional SNP, located proximally to the index SNP rs4792887, as well as with two distal SNPs, which were correlated with index SNP rs4792887.
		rs110402	Intron	C > T							
		rs12936511	Exon	C > T							
		rs242939	Intron	A > G							
		rs242938	Intron	A > C							
		rs1876831	Intron	C > T							
		rs16940665	Exon	T > C							
		rs4792887	Intron	C > T							
		rs110402	Intron	C > T							
De Luca, V. 2010 [27]	17	rs16940665	Exon	T > C	SZ	SA	81	150	-	Blood	No association.
Ben-Efraim, Y. J. 2011 [46]	17	rs4792887	Intron	C > A	DE	SA	284	354	SLEI	-	1. G×E predominantly in females with SA between rs7209436 and childhood/adolescence physical assault or attack. 2. Male-specific G×E between rs16940665 and physical assault or attack exposure in adulthood. 3. Male-specific G×E in depressed SA, rs4792887, and cumulative stressful life events.
		rs110402	Intron	C > T							
		rs16940665	Exon	T > C							
		rs4792887	Intron	C > T							
Roy, A. 2012 [28]	17	rs9900679	Intron	A > C	SUD	SA	141	689	CTQ	Blood	No association.
		rs4792887	Intron	C > T							
		rs110402	Intron	C > T							
		rs249224	Intron	C > A							
		rs8072451	Intron	C > T							
		rs81189	Intron	G > C							
		rs24939	Intron	A > G							
		rs173365	Intron	T > C							
		rs17689918	Intron	G > A							
Guillaume, S. 2013 [47]	17	rs242948	Downstream	C > T	Mixed	SA	218	-	CTQ	Blood	Sexual abuse and emotional neglect in childhood interacted with rs1396862, rs878886, and rs242948 to modulate adult decision making in SA.
		rs1396862	Intron	G > A							
		rs878886	3′UTR	G > T							
		rs4076452	Intron	G > C							
Leszczynska-Rodziewicz, A. 2013 [48]	17	rs4076452	Intron	G > C	BD	SA	225	712	-	Blood	No association.
		rs12936511	Exon	C > T							
		rs4792887	Intron	C > T							
		rs24290	Intron	T > C							
		rs878886	3′ UTR	G > T							
		rs173365	Intron	T > C							
		rs110402	Intron	C > T							
Breen, M. E. 2015 [38]	17	rs2664008	Intron	G > A	BD	SA	631	657	ELES	-	Significant interaction between rs2664008 and a history of childhood physical and/or sexual abuse was reported; however, this interaction was not significant after correcting for multiple testing.
		rs1724425	Intron	C > T							
		rs1526123	Intron	T > A							
		rs6593447	Intron	A > G							
		rs11655764	Intron	G > A							
Pawlak, J. 2016 [49]	17	rs4792877	Intron	A > G	AD	SA	277	847	-	Blood	rs16940665 polymorphism was associated with SA in MDD males.
		rs12936511	Exon	C > T							
		rs110402	Intron	C > T							
		rs16940665	Exon	T > C							
Mirkovic, B. 2017 [42]	17	rs4792887	Intron	C > T	Mixed	SA	98	150	-	Buccal	No association.
Bastos, C. R. 2017 [50]	17	rs110402	Intron	C > T	Mixed	SI and SA	SI: 15SA: 20	136	-	Blood	Individuals who carried the A allele increased in 15% additional risk for SA via the increase in IL-1b levels.
Ludwig, B. 2018 [51]	17	rs7209436	Intron	C > T	AD	SA	70	181	CTQ and BLEQ	Blood	Significant gene-environment-interactions were found for the SNPs rs7209436 and rs110402, reflecting the impact of childhood trauma and CRHR1 gene polymorphisms in previous SA.
		rs4792887	Intron	C > T							
		rs110402	Intron	C > T							
		rs242924	Intron	C > A							
		rs242939	Intron	A > G							
Segura, A. G. 2019 [19]	17	rs110402	Intron	C > T	BD	SB	129	-	CTQ	Blood	No association.
		rs242940	Intron	A > G							
Sanabrais-Jiménez, M.A. 2019 [23]	17	rs110402	Intron	C > T	BD and MDD	SA	183	183	CTQ	Blood	The analysis showed an interaction of *CRHR1* and *CRHR2* genes with childhood trauma, thus conferring increased risk of having presented at least one SA.
		rs242924	Intron	C > A							
		rs16940665	Exon	T > C							
Nobile, B. 2020 [44]	17	rs878886	3′UTR	G > T	MDD	SI and SA	SI: 99SA: 9	384	-	Buccal	No association.
*CRHR2*											
Papiol, S. 2007 [31]	7	rs2240403	Exon	C > T	MDD	*SA*	24	96	*-*	Blood	Allele G carriers of rs2270007 showed a worse overall response to citalopram though time of follow-up and showed 2.93 increased risk for nonresponding to citalopram treatment at week 4.
		rs2270007	Intron	G > C							
De Luca, V. 2010 [27]	7	rs1076292	Intron	C > T	SZ	SA	81	150	Blood	No association	No association.
Roy, A. 2012 [28]	7	rs3779250	Intron	G > A	SUD	SA	141	689	CTQ	Blood	No association.
		rs973002	Intron	A > G							
		rs8192498	-	G > A							
		rs2190242	Intron	A > C							
		rs2284217	Intron	G > A							
		rs2014663	Intron	A > G							
		rs6967702	5′ region	G > C							
		rs4723002	Intergenic	A > G							
		rs255102	Intergenic	T > A							
		rs255105	Intergenic	T > C							
		rs255125	Intergenic	G > A							
Guillaume, S. 2013 [47]	7	rs255098	Intron	G > A	Mixed	SA	218	-	CTQ	Blood	Sexual abuse and emotional neglect in childhood interacted with rs255098 to modulate adult decision making in SA.
		rs2270007	Intron	G > C							
Breen, M. E. 2015 [38]	7	rs2267716	Intron	T > C	BD	SA	631	657	ELES	-	No association.
		rs11980048	Intron	G > T							
		rs4723002	Intron	A > G							
		rs2190242	Intron	C > A							
		rs4723003	Intron	C > T							
Segura, A. G. 2019 [19]	7	rs4722999	Intron	C > T	BD	SB	129	-	CTQ	Blood	No association.
		rs2284219	Intron	A > G							
		rs255115	Intron	G > A							
		rs255102	Intergenic	T > A							
Sanabrais-Jiménez, M.A. 2019 [23]	7	rs2190242	Intron	C > A	BD and MDD	SA	183	183	CTQ	Blood	An interaction of *CRHR1* and *CRHR2* genes with childhood trauma, thus conferring an increased risk of having presented at least one SA.
		rs2284217	Intron	G > A							
		rs2014663	Intron	A > G							
*NR3C1*											
De Luca, V. 2010 [27]	5	rs6196	Exon	A > G	SZ	SA	81	150	-	Blood	This SNP was significantly associated with SA, positively protecting against suicide attempt.
Supriyanto, I. 2011 [36]	5	rs6196	Exon	A > G	-	CS	219	228	-	Blood	No association.
		rs10052957	Intron	G > A							
Leszczynska-Rodziewicz, A. 2013 [48]	5	rs41423247	Intron	G > C	BD	SA	225	712	-	Blood	No association.
		rs6195	Intron	T > C							
		rs6198	3′UTR	T > C							
		rs6191	3′UTR	C > A							
		rs6196	Exon	A > G							
		rs33388	Intron	A > G							
Breen, M. E. 2015 [38]	5	rs4912905	Intron	G > C	BD	SA	631	657	ELES	-	No association.
		rs10042042	Intron	G > A							
		rs17209251	Intron	A > G							
		rs17100236	Intron	T > C							
		rs10477211	Intron	A > G							
Yin, H. 2016 [41]	5	rs6196	Exon	A > G	MDD	SA and CS	SA: 87	190	-	SA: -	rs9324924 showed evidence of association at uncorrected *p* < 0.05 level with SA.
		rs33388	Intron	A > C							
		rs33389	Intron	C > T			SV: 121	88		SV: Brain	
		rs10052957	Intron	G > A							
		rs9324924	Intron	G > A							
Park, S. 2016 [20]	5	rs41423247	Intron	G > C	Cancer	CS	182	161	-		SNP was associated with the susceptibility to suicide within the first year after cancer diagnosis.
Segura, A. G. 2019 [19]	5	rs6198	3′UTR	T > C	BD	SB	129	-	CTQ	Blood	No association.
		rs2963156	Intron	T > C							
		rs1837262	Intron	C > A							
		rs4912910	Intron	A > G							
		rs4634384	Intron	C > T							
Nobile, B. 2020 [44]	5	rs2963155	Intron	A > G	MDD	SI and SA	SI: 99	384	-	Buccal	AG genotype of rs2963155 was associated with SI. This association was not significant after multiple test correction.
		rs33388	Intron	A > C							
		rs4912905	Intron	G > C							
		rs41423247	Intron	G > C			SA: 9				
		rs6189	Exon	C > T							
		rs12656106	Intron	G > C							
		rs4607376	Intron	G > T							
*NR3C2*											
Supriyanto, I. 2011 [36]	4	rs5525	Exon	A > C	CS	CS	219	228	-	Blood	No association.
		rs5522	Exon	C > T							
		rs2070951	5′UTR	G > A							
Segura, A. G. 2019 [37]	4	rs5534	3′UTR	T > C	BP	SB	129	-	CTQ	Blood	No association.
		rs12499208	Intron	T > C							
		rs6846591	Intron	T > C							
		rs5522	Exon	C > T							
*SKA2*											
Kaminsky, Z. 2015 [25]	17	rs7208505	3′UTR	G > T	PTSD	SI and SA	SI: 325 SA: 746	658	CTQ	Blood and Saliva	Significant interactions of SKA2 3′-UTR DNA methylation and rs7208505 genotype for SI and SA.
Yin, H. 2016 [41]	17	rs8082544	-	A > G	MDD	SA and CS	SA: 87SV: 121	190 88	-	SA: - SV: Brain	1. rs12945875, rs9911583, and rs8067682 showed evidence for association at uncorrected *p* < 0.05 level with SA. 2. rs8082544 and rs7502947 showed association with death by suicide.
		rs12945875	Intron	G > A							
		rs9911583	Intron	G > A							
		rs8067682	Intron	A > G							
		rs7502947	Intron	G > A							
Sadeh, N. 2016 [52]	17	rs7208505	3′UTR	G > T	PTSD	SI, SP, SA	SI: 146 SA: 50 SP:92	-	-	Blood	No association.
Nobile, B. 2020 [44]	17	rs7208505	3′UTR	G > T	MDD	SI and SA	SI: 99 SA:9	384	-	Buccal	GG/AG genotype was significantly associated with SI. This association was not significant after multiple test correction.
*MC2R*											
De Luca, V. 2010 [27]	18	rs4797825	3′UTR	C > T	SZ	SA	81	150	-	Blood	No association.
Breen, M. E. 2015 [38]	18	rs3744819	3′UTR	C > A	BD	SA	631	657	ELES	-	No association.
		rs12456733	Intron	G > A							
		rs1941088	Intron	G > A							
		rs3888305	3′UTR	A > C							
		rs4308014	3′UTR	C > T							
Segura, A. G. 2019 [37]	18	rs4797825	3′UTR	C > T	BD	SB	129	-	CTQ	Blood	No association.
		rs9961110	Intron	T > C							
		rs17624314	Intron	A > G							
*POMC*											
Breen, M. E. 2015 [38]	2	rs7565877	intron	A > G	BD	SA	631	657	ELES	-	No association.
		rs6545975	intron	C > A							
		rs7565427	intron	A > C							
		rs934778	intron	A > G							
		rs1866146	Downstream	G > A							
Segura, A. G. 2019 [19]	2	rs713586	Intron	T > C	BD	SB	129	-	CTQ	Blood	No association.
		rs6713532	Intron	T > C							
		rs6545975	Intron	C > A							
		rs934778	Intron	A > G							

AD, alcohol-dependent; BD, bipolar disorder; DE, depression; MDD, major depression disorder; PTSD, post-traumatic stress disorder; SA, suicide attempt; SB, suicide behavior; SI, suicide ideation; SP, suicide plan; CS, completed suicide; SUD, substance use dependence; SD, schizoaffective disorder; SZ, schizophrenia; BLEQ, Brief Life Events Questionnaire; ELES, Early Life Events Scale; ACE, Adverse Childhood Experiences Questionnaire; CTQ, Childhood Trauma Questionnaire; LEC, Life Events Checklist; SLEI, Stressful Life Event Inventory.

**Table 2:** Characteristics of the included publications that evaluated HPA genes expression (mRNA) in the pathogenesis of suicide behavior.

Author, Year	Suicide Behavior	N	Ethinicity	Tissue Source	Expression	Variant	Genotype/Expression	Trauma Exposure	Cortisol Concentrations
*NR3C1*									
McGowan, P. 2009 [53]	CS	CS: 24 Controls:12	Caucasian	Brain	↓	-	-	mRNA was significantly reduced in SV with a history of childhood abuse relative to non-abused SV	-
Sinclair, D. 2012 [54]	CS	CS: 21, Controls: 34	Caucasian	Brain	↓	rs10052957 rs72801094 rs5871845 rs10482614 rs10482616 rs4634384 rs6190 rs1800445 rs41423247 rs6196 rs6198	rs10052957, rs6190, rs41423247 ↓	-	-
Pérez-Ortiz, J. M. 2013 [14]	CS	CS: 13 Controls: 13	Caucasian	Brain	↓	-	-	-	-
Zhao, J. 2015 [55]	CS	CS: 17 Controls: 7	Caucasian	Brain	No changes	-	-	-	-
Yin, H. 2016 [41]	CS	CS: 21, Controls: 38	European	Brain	↓	rs6196 rs33388 rs33389 rs10052957 rs9324924	No association.	-	-
Roy, B. 2017 [56]	SI	SI: 14, Controls: 20	Caucasian and African-American	Blood	↓	-	-	-	-
Melhem, N. M. 2017 [57]	SA and SI	SA:38, SI:40, Controls:37	Caucasian	Blood	SA ↓	-	-	mRNA was significantly and negatively associated with childhood abuse.	HCC was associated with mRNA.
Chang, H. B. 2019 [58]	SA and SI	SA:38, SI:40, Controls:37	Caucasian	Blood	No changes	-	-	-	No association.
*FKBP5*									
Pérez-Ortiz, J. M. 2013 [14]	CS	CS: 13 Controls: 13	Caucasian	Brain	↓	-	-	-	-
Yin, H. 2016 [41]	CS	CS: 21 Controls: 38	European	Brain	↓	rs3800373 rs9296158 rs3777747 rs4713902 rs9470080 rs7757037 rs737054 rs9380529	No association	-	-
Roy, B. 2017 [56]	SI	SI: 14 Controls: 20	Caucasian and African-American	Blood	↓	-	-	-	-
Melhem, N. M. 2017 [57]	SA and SI	SA:38; SI:40 Controls:37	Caucasian	Blood	SI ↓	-	-	No association.	No association.
Chang, H. B. 2019 [58]	SA and SI	SA:38; SI:40 Controls:37	Caucasian	Blood	mRNA was consistently correlated with heroin, painkillers, and ecstasy use.	-	-	-	No association.
*CRHR1*									
Hiroi, N. 2001 [59]	CS	CS: 9 Controls: 7	Caucasian	Brain	No change	-	-	-	-
Merali, Z. 2004 [60]	CS	CS: 12 Controls: 12	Caucasian	Brain	↓	-	-	-	-
Zhao, J. 2015 [55]	CS	CS: 17 Controls: 7	Caucasian	Brain	No change	-	-	-	-
Roy, B. 2017 [56]	CS	CS: 14 Controls: 20	Caucasian and African-American	Blood	No change	-	-	-	-
*CRHR2*									
Hiroi, N. 2001 [59]	CS	CS: 9 controls: 7	Caucasian	Pituitary	No change	-	-	-	-
Merali, Z. 2004 [60]	CS	CS: 12 Controls: 12	Caucasian	Brain	No change	-	-	-	-
Zhao, J. 2015 [55]	CS	CS: 17 Controls: 7	Caucasian	Brain	No change	-	-	-	-
*CRHBP*									
Merali, Z. 2004 [60]	CS	CS: 12 Controls: 12	Caucasian	Brain	No change	-	-	-	-
Zhao, J. 2015 [55]	CS	CS: 17 Controls: 7	Caucasian	Brain	No change	-	-	-	-
Roy, B. 2017 [56]	SI	SI: 14 Controls: 20	Caucasian and African-American	Blood	No change	-	-	-	-
*CRH*									
Merali, Z. 2004 [60]	CS	CS: 12 Controls: 12	Caucasian	Brain	↑	-	-	-	-
Zhao, J. 2015 [55]	CS	CS: 17 Controls: 7	Caucasian	Brain	↑	-	-	-	-
*SKA2*									
Yin, H. 2016 [41]	CS	CS: 21 Controls: 38	European	Brain	↓	rs8082544	AG ↓	-	-
						rs7502947	AG ↓		
Pandey, G.N 2016 [26]	CS	CS: 52 Control: 51	Caucasian	Brain	↓	-	-	-	
Melhem, N. M. 2017 [57]	SA and SI	SA:38, SI:40, Controls:37	Caucasian	Blood	SI ↑	-	-	No association.	No association.
Chang, H. B. 2019 [58]	SA and SI	SA:38, SI:40, Controls:37	Caucasian	Blood	Not changes	-	-	-	No association.

↑, high expression; ↓, reduced expression; SA, suicide attempt; SI, suicide ideation; CS, completed suicide; HCC, hair cortisol concentration.

**Table 3:** NOS scores of 41 studies included in the systematic review.

Study	Year	Selection	Comparability	Outcome/Exposure	Score
Hiroi, N. [59]	2001	★★★	★ ★	★★	7
Merali, Z. [60]	2004	★★★	★ ★	★★	7
Papiol, S. 2007 [31]	2007	★★	★ ★	★★	6
Wasserman, D. [45]	2008	★★	★ ★	★ ★ ★	7
Willour, V. L. [32]	2009	★★★	★ ★	★★	7
Wasserman, D. [24]	2009	★★	★ ★	★ ★ ★	7
McGowan, P. [53]	2009	★★	★ ★	★ ★ ★	7
De Luca, V. [27]	2010	★★★★	★ ★	★★	8
Brent, D. [33]	2010	★★★	★ ★	★★	7
Roy, A. [34]	2010	★★★★	★ ★	★★	8
Perroud, N. [35]	2011	★ ★	★ ★	★ ★	6
Supriyanto, I. [36]	2011	★ ★ ★ ★	★ ★	★ ★	8
Ben-Efraim, Y. J. [46]	2011	★ ★ ★	★ ★	★★	7
Sinclair, D. [54]	2012	★ ★	★ ★	★★	6
Roy, A. [28]	2012	★★★	★ ★	★ ★	7
Guillaume, S. [47]	2013	★ ★	★ ★	★ ★	6
Leszczynska-Rodziewicz, A. [48]	2013	★ ★	★ ★	★ ★	6
Pérez-Ortiz, J. M. [14]	2013	★ ★ ★ ★	★ ★	★ ★	8
Leszczynska-Rodziewicz, A. [37]	2014	★ ★	★ ★	★ ★	6
Zhao, J. [55]	2015	★★	★ ★	★ ★	6
Breen, M. E. [38]	2015	★★★	★ ★	★★	7
Fudalej, S. [39]	2015	★★	★ ★	★★	6
Kaminsky, Z. [25]	2015	★★★	★ ★	★★	7
Park, S. [20]	2016	★★★	★ ★	★★	7
Breen, M. E. [40]	2016	★★★★	★ ★	★★	8
Pawlak, J. [49]	2016	★★★★	★ ★	★★	8
Pandey, G.N [26]	2016	★★★★	★ ★	★★ ★	9
Sadeh, N. [52]	2016	★ ★	★ ★	★ ★	6
Yin, H. [41]	2016	★★★★	★ ★	★★	8
Mirkovic, B. [42]	2017	★★★	★ ★	★★★ ★	9
Roy, B. [56]	2017	★★★	★ ★	★★	7
Bastos, C. R. [50]	2017	★★★★	★ ★	★★★	9
Melhem, N. M. [57]	2017	★★★	★ ★	★★★	8
Ludwig, B. [51]	2018	★ ★	★ ★	★★★	7
Chang, H. B. [58]	2019	★★	★ ★	★★★	7
Sanabrais-Jiménez, M.A. [23]	2019	★★	★ ★	★★	6
Segura, A. G. [19]	2019	★★	★ ★	★★	6
Zhang, L. [43]	2019	★★★	★★	★★	7
Nobile, B. [44]	2020	★★★	★ ★	★★	7
Berent, D. [21]	2020	★★★★	★ ★	★★ ★	9
Hernández-Díaz, Y. [22]	2021	★★★	★ ★ ★	★★ ★	9

The NOS scale range from zero to nine, giving a point (star) to each accomplished item, categorizing the studies as high quality (score 7–9), moderate quality (score 4–6), or poor quality (score 0–3).

## Data Availability

Not applicable.

## Supplementary Materials

The following are available online at https://www.mdpi.com/article/10.3390/genes12101608/s1, Table S1: PRISMA checklist.
